# Soil Fungal Community Structure in Boreal Pine Forests: From Southern to Subarctic Areas of Finland

**DOI:** 10.3389/fmicb.2021.653896

**Published:** 2021-05-26

**Authors:** Zhao-Lei Qu, Minna Santalahti, Kajar Köster, Frank Berninger, Jukka Pumpanen, Jussi Heinonsalo, Hui Sun

**Affiliations:** ^1^Collaborative Innovation Center of Sustainable Forestry in Southern China, College of Forestry, Nanjing Forestry University, Nanjing, China; ^2^Faculty of Agriculture and Forestry, Institute for Atmospheric and Earth System Research/Forest Sciences, University of Helsinki, Helsinki, Finland; ^3^Department of Environmental and Biological Science, University of Eastern Finland, Kuopio, Finland; ^4^Department of Microbiology, Faculty of Agriculture and Forestry, University of Helsinki, Helsinki, Finland

**Keywords:** boreal forest, Scots pine, fungal community structure, community potential function, geographical location

## Abstract

The boreal forest environment plays an important role in the global C cycle due to its high carbon storage capacity. However, relatively little is known about the forest fungal community at a regional scale in boreal forests. In the present study, we have re-analyzed the data from our previous studies and highlighted the core fungal community composition and potential functional groups in three forests dominated by Scots pine (*Pinus sylvestris* L.) in Finland, and identified the fungal generalists that appear across geographic locations despite differences in local conditions. The three forests represent subarctic, northern and southern boreal forest, and are all in an un-managed state without human interference or management. The subarctic and northern areas are subject to reindeer grazing. The results showed that the three locations formed distinct fungal community structures (*P* < 0.05). Compared to the two northern locations, the southern boreal forest harbored a greater abundance of Zygomycota, *Lactarius*, *Mortierella Umbelopsis*, and *Tylospora*, in which aspect there were no differences between the two northern forests. *Cortinarius*, *Piloderma*, and *Suillus* were the core fungal genera in the boreal Scots pine forest. Functionally, the southern boreal forest harbored a greater abundance of saprotroph, endophytes and fungal parasite-lichen, whereas a greater abundance of ectomycorrhizal fungi was observed in the northern boreal forests. Moreover, the pathotroph and wood saprotrophs were commonly present in these three regions. The three locations formed two distinct fungal community functional structures, by which the southern forest was clearly separated from the two northern forests, suggesting a distance–decay relationship via geographic location. This study provides useful information for better understanding the common fungal communities and functions in boreal forests in different geographical locations.

## Introduction

Microorganisms, including bacteria, archaea and fungi, play a pivotal role in forest ecosystems (Ma et al., 2017; Morriën et al., 2017), as they are involved in organic matter decomposition and nutrient cycles (Warcup, 1951). For example, some nitrogen-fixing bacteria can help plant for nitrogen uptake and participant in the nitrogen cycle (Falkowski et al., 2008), which is especially important in N-poor boreal forests due to low organic decomposition rate (Högberg et al., 2017). Some fungi can act as decomposers, mutualists, and pathogens, influentially coexisting with plants (Dantas and Sommer, 2014). In N-limited boreal forests, mycorrhizal fungi could affect tree C allocation and retain much N and transfer little to trees (Högberg et al., 2017). The soil microbes interact with below- and above-ground parts of plants to maintain the stability of the forest ecosystem. The soil microbial community is more prone to being affected by changes in regional or local conditions than plants (Fierer and Jackson, 2006; Bissett et al., 2010). Many studies have focused on microbial communities at a large scale globally or large areas from the southern to the northern regions of China (Tedersoo et al., 2014; Ma et al., 2016), however, it is not easy to elucidate the effects of abiotic factors on the microbial community as such scale covering large span of climate and environmental conditions. Therefore, understanding the microbial community structure and ecological function at the regional or local scale can help to better understand the responses of microbes to changes in environmental and climatic conditions.

Boreal forests are characterized by high carbon storage capacities in soils due to the accumulation of plant litter and humic materials (Berg, 2009), which play important roles in the global carbon cycle (Goodale et al., 2002; Magnani et al., 2007). The Finnish boreal forests, being like most of the other boreal forests, are N-poor soils, in which strong N retention by microorganisms keeps levels of available N very low (Heinonsalo et al., 2015; Högberg et al., 2017). The microbial communities in Finnish boreal forests have been investigated in terms of temporal and spatial changes (Santalahti et al., 2016), disturbance by fire (Sun et al., 2015), reindeer gazing (Santalahti et al., 2018), and their relationships with the dominant tree species (Sun et al., 2016). Most of the abovementioned studies were confined to a single local Finnish site. Relatively little is known about these microbial communities at a regional scale in Finland. Knowledge of microbial communities at a regional scale could help us to better understand the function of the entire boreal forest.

Soil fungi are a highly diverse component of soil microbial communities (Wu et al., 2013), which have the ability to decompose and mineralize complex recalcitrant compounds of plant origin, such as cellulose, hemicellulose and lignin (Orgiazzi et al., 2012). Soil saprotrophs can participate in nutrient recycling as decomposers (Mäkipää et al., 2017). Soil fungi can also interact with plant as mutualists and pathogens (Peay et al., 2016). Symbiotic fungi are of crucial importance in facilitating nutrient uptake for plant growth in most forest ecosystems (Smith and Read, 2008; Talbot et al., 2013). Pathogenic fungi typically have a negative regulatory effect on plant growth, and can change the plant community’s diversity and composition (Bagchi et al., 2014). It has been suggested that fungi may be more influential than bacteria in terms of the ecological function of the boreal forest ecosystem (Clemmensen et al., 2013). One of the reasons for this might be the low pH and high carbon content in boreal forests (Delgado-Baquerizo and Eldridge, 2019), which together result in the low bacterial diversity (Rousk et al., 2010). The higher contents of recalcitrant carbon compounds in boreal forests may also hinder bacterial activity, as they give fungi an advantage in decomposition (Rousk et al., 2016; Treseder et al., 2016). Consequently, exploring the impacts of local variables on the fungal community’s structure can help us to better understand the ecological functions of fungi in different niches.

Our previous studies have investigated the spatial and temporal changes of soil fungal communities in the southern boreal forest (Hyytiälä) (Santalahti et al., 2016), and the effects of reindeer grazing on the soil fungal community in the northern boreal forest (Sodankylä) and subarctic boreal forest (Värriö) (Santalahti et al., 2018). The sothern forest is in its natural state, whereas, the northern and subarctic forests have been undergoing reindeer grazing naturally. However, the three forests have similar histories with un-managed conditions and are all dominated by Scots pine (*Pinus sylvestris* L.). In the present study, we conduct an integrated re-analysis on raw data retrieved from our previously published results on three different locations in southern boreal (Hyytiälä), northern boreal (Sodankylä) and subarctic (Värriö) Scots pine (*P*. *sylvestris* L.) forests (Santalahti et al., 2016, 2018). The high throughput sequence (HTS) methods used in these studies were all 454 pyrosequencing by using the same fungal primer pairs targeting the same fungal region of internal transcribed spacer 2 (ITS2), which enable the comparison of results obtained from HTS in different studies. To ensure analysis accurancy, only the samples with similar sampling treatments (e.g., sampling period, sampling location) in each forest were selected. Therefore, the main aim of the study was to identify the difference in fungal communities compositions and potential functional groups between southern and northern boreal forests in Finland. Additionally, we aimed to identify some fungal potential generalists across different locations. Previous studies have shown that different tree species and vegetations can shift soil microbial community in boreal forests (Chu et al., 2011; Sun et al., 2016). The forests with same dominant tree species might harbor some similar microbes. Our hypothesis is that there might be a core fungi community (a fungal community that exists stably and is not affected by geographical factors), or fungal generalists, in boreal pine forests across different geographic locations, despite differences in local conditions.

## Materials and Methods

### Sampling Sites and Sample Property

The southern, northern and subarctic boreal forests are located in Hyytiälä (61°51′N, 24°17’E), Sodankylä (67°21′N, 26°38′E), and Värriö Strict Nature Reserve (67°46’N, 29°35′E), respectively (Supplementary Table 1). Information on the soil properties and the aboveground vegetation in northen (Sodankylä) and subarctic (Värriö) forests is presented in Supplementary Table 2.

In the present study, in order to ensure consistency with the sampling periods and soil horizons across the three forests, only samples from the humus horizon collected in June and July in 2011 in southern forest were selected for analysis. Since grazing is the natural use-condition of the forest vegetation in northern and subarctic areas, only samples from the humus horizon collected in June 2013 in these two forests were selected and included in the analysis.

### Sample Selection and Raw Sequences Retrieval

The fungal community analysis included three locations, with 21 samples in total. The southern boreal forest site provided six samples, with three samples each from June and July, and only samples from the lower organic horizons were included (Santalahti et al., 2016). The sequences of each sample were obtained by combining sequences from the fragmented litter O/F and the humus O/H. The northern boreal forest in Sodankylä provided five soil samples, all from the grazed area, for analysis (Santalahti et al., 2016). The subarctic boreal forest in Värriö provided ten samples in total (Santalahti et al., 2016), of which four were from the grazed area in Kotovaara and six were from the grazed area in Nuortti. More details on the sampling process are available in Santalahti et al. (2016, 2018).

The methods used in each study for DNA extraction, primer pairs (gITS7 and ITS4) and high-throughput sequencing (454 pyrosequencing) were identical, and have been described in detail by Santalahti et al. (2016, 2018). Raw reads from the selected samples from each of the previous studies were retrieved from the European Nucleotide Archive at the European Bioinformatics Institute with the accession codes PR-JEB21587^1^ and PRJEB10726^2^.

The selected raw sequences used in this study are available from the Sequence Read Archive (SRA) of the National Center for Biotechnology Information (NCBI) under project accession number PRJNA703504.

### Re-analysis of Pyrosequence Data

The raw sequences were denoised and quality-controlled following the standard operating procedure (SOP) using Mothur (v. 1.39.5) (Schloss et al., 2011; Heinonsalo et al., 2015). Sequences were removed if they contained any of the following: (i) ambiguous (N) bases; (ii) homopolymers longer than eight nucleotides; (iii) an average quality score lower than 25; (iv) chimeras (detected using Chimera uchim in Mothur); (v) fewer than 200 nucleotides. The remaining sequences were pre-clustered within a distance of 1 bp using a pseudo-single-linkage algorithm implemented in Mothur, so as to minimize the number of sequences that arose due to pyrosequencing errors (Huse et al., 2010). All potentially chimeric sequences were identified and removed by the Mothur-embedded UCHIME program (Edgar et al., 2011). Unique sequences were pairwise aligned via the Needleman method (Needleman and Wunsch, 1970). The aligned sequences were then clustered into operational taxonomic units (OTUs), using the average neighbor-joining algorithm, at 97% similarity, and the OTUs containing only one sequence across all the samples were omitted (Tedersoo et al., 2010). The sequences were assigned to taxonomic groups with 80% bootstrap confidence using the naïve Bayesian classifier (Wang et al., 2007) and the mothur-formatted UNITE taxonomy reference (UNITE+INSD, version 8). The diversity indices including observed and estimated species richness (Chao1) (Chao, 1984), diversity [inverse Simpson’s index (1/D)], and evenness [Simpson’s equitability (ED)] were estimated (Schloss et al., 2009). FUNGuild was used to identify fungal functional guilds and determine the trophic modes (symbiotroph, saprotroph, and pathotroph) via the annotation of ITS sequence classifications, through which the three trophic modes were further divided into 15 functional groups at three levels of credibility (“probable,” “highly probable,” and “possible”) (Nguyen et al., 2016).

Analysis of variance (ANOVA) was used to test the significant difference (*P* < 0.05) in species richness, alpha diversity, evenness, and the abundance of taxonomic groups, using the software IBM SPSS Statistic 22 (Davies and Gray, 2015). Statistical analysis of metagenomic profiles (STAMP)was used to identify the functional groups showing significant differences between locations (Parks et al., 2014). Canonical analysis of principal (CAP) coordinates, and the PERMANOVA package in PRIMER 7 were used to visualize the community’s structure based on the OTU abundance (Anderson et al., 2008). CAP analyses were based on the Bray-Curtis dissimilarity measure of log-transformed abundance of OTUs on each replicate. The number of permutations in the CAP analyses was set to 100, which allowed to select the optimal number of meaningful PCO axes (m) required to provide the best distinction between sites. The first two axes (CAP1 and CAP2), which explained most of the variation, were used to construct ordination plots. Venn diagrams were drawn to distinguish the shared and unique OTUs among sites. Distance-based linear models (DistLM) was used to test the correlations between the top 10 most abundant fungal species and community or functional structures. Both Venn diagrams and DistLM were carried out in PRIMER 7 (Anderson et al., 2008).

## Results

### Information on Re-analyzed Data

The 454 pyrosequencing of 21 samples from three locations resulted in a total of 186,138 sequences after quality control, which were included in the analysis. The number of sequences in each sample ranged from 2,758 to 21,454, with an average of 8,864 ± 5,063 (mean ± SD). The sequences were assigned across 1,064 OTUs and the rarefaction curve indicating the sequences depth was shown in Supplementary Figure 1.

### Fungal Community Diversity in the Three Locations

**FIGURE 1:**
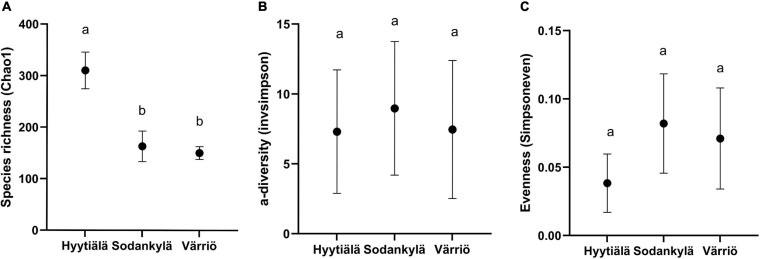
The fungal community **(A)** species richness (Chao 1), **(B)** diversity [inverse Simpson’s index (1/D)], and **(C)** evenness [Simpson’s equitability (ED)] in the three locations between Hyytiälä (southern), Sodankylä (northern) and Värriö (subarctic) boreal pine forests. Values in the figure was shown by the mean with standard deviation (*n* = 6 for Hyytiälä, *n* = 5 for Sodankylä, and *n* = 10 for Värriö) and letters (a and b) in the figure showing the significance at 95% confident level between locations.

The fungal species richness (Chao 1) was significantly higher in the southern boreal forest (in Hyytiälä) compared to the northern and subarctic boreal forests (in Sodankylä and in Värriö) (*P* < 0.05) (Figure 1A). The diversity (1/D) and evenness (ED) did not differ between the three locations. Hyytiälä displayed the least fungal evenness, showing no differences in diversity between the three locations (Figures 1B,C).

### Fungal Community Composition at the Taxonomic Level in the Three Locations

**FIGURE 2:**
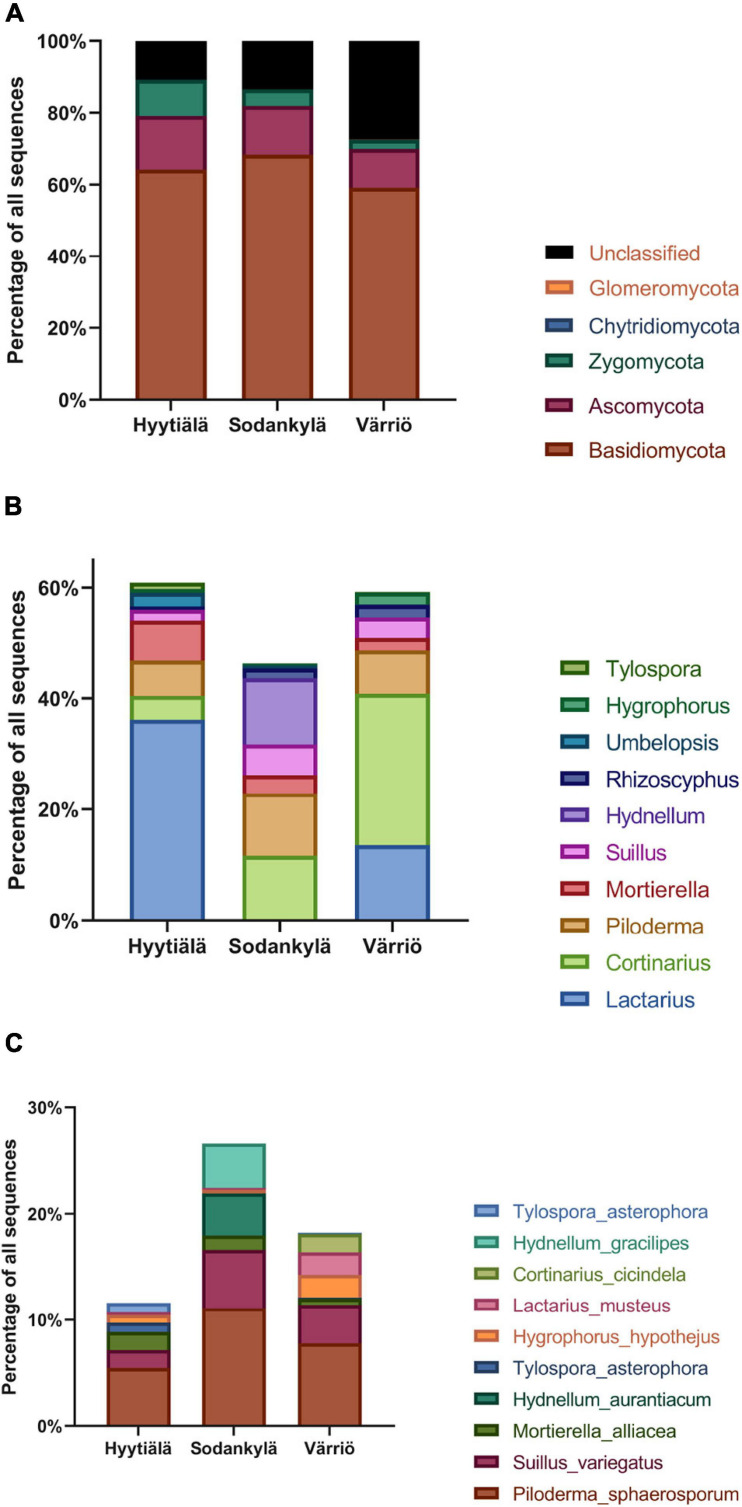
The fungal abundance at different taxonomic levels. **(A)** Phylum, **(B)** genus (the top 10 most abundant), and **(C)** species (the top 10 most abundant) in the three locations between Hyytiälä (southern), Sodankylä (northern) and Värriö (subarctic) boreal pine forests.

The 1,065 OTUs were assigned to five phyla, accounting for 84.8% of the total sequences. Basidiomycota were the most abundant phyla in all areas, accounting for 66.5% of the total sequences, followed by Ascomycota (12.2%), Zygomycota (6.1%), Glomeromycota (0.03%), and Chytridiomycota (0.01%) (Figure 2A and Supplementary Figure 2). Only Zygomycota showed significantly greater abundance in the southern boreal forest compared to the northern or subarctic boreal forests (*P* < 0.05) (Figure 2A).

In our results, 65.4% of the total sequences were further classified into 90 genera, which ranged from 30.9 to 84.0% in terms of proportion of the total sequences in the different locations. The top 10 most abundant genera accounted for 60.6% of the total sequences. *Lactarius* (21.6%) was the most abundant genus, followed by *Cortinarius* (18.3%), *Piloderma* (6.7%), *Mortierella* (4.5%), *Suillus* (3.8%), *Hydnellum* (2.0%), *Rhizoscyphus* (1.3%), *Umbelopsis* (1.1%) *Hygrophorus* (0.7%), and *Tylospora* (0.6%). The most abundant genera in each location differed. *Lactarius*, *Mortierella*, and *Piloderma* were the dominant genera in Hyytiälä. *Cortinarius*, *Piloderma*, and *Hydnellum* were the dominant genera in Sodankylä, whereas *Lactarius*, *Cortinarius*, and *Piloderma* were most abundant in Värriö (Figure 2B). Among the most dominant genera in each location (the top 10 genera), *Cortinarius*, *Piloderma*, and *Suillus* were the most abundant genera in the three locations, and geographic differences did not affect their abundances. Moreover, *Lactarius*, *Mortierella Umbelopsis*, and *Tylospora* were more abundant in Hyytiälä than in Sodankylä and Värriö (*P* < 0.05), whereas *Hydnellum* were more abundant in Sodankylä than in Hyytiälä and Värriö (*P* < 0.05).

The sequences were classified into 118 species, accounting for 20.5% of the total sequences (ranging from 2.5 to 43.3% of the sequences in different locations). The most abundant species across the three locations was *Piloderma sphaerosporum*, accounting for 6.1% of the sequences, followed by *Suillus variegatus*, *Mortierella alliacea, Hydnellum aurantiacum, Hygrophorus hypothejus, Lactarius musteus Cortinarius cicindela*, and *Hydnellum gracilipes*. Hyytiälä harbored the highest levels of *Mortierella alliacea*, compared to Värriö (Figure 2C). *Piloderma sphaerosporum*, *Suillus variegates*, and *Hygrophorus hypothejus* showed similar abundances across the three locations.

### Fungal Community Composition at the OTUs Level in the Three Locations

**FIGURE 3:**
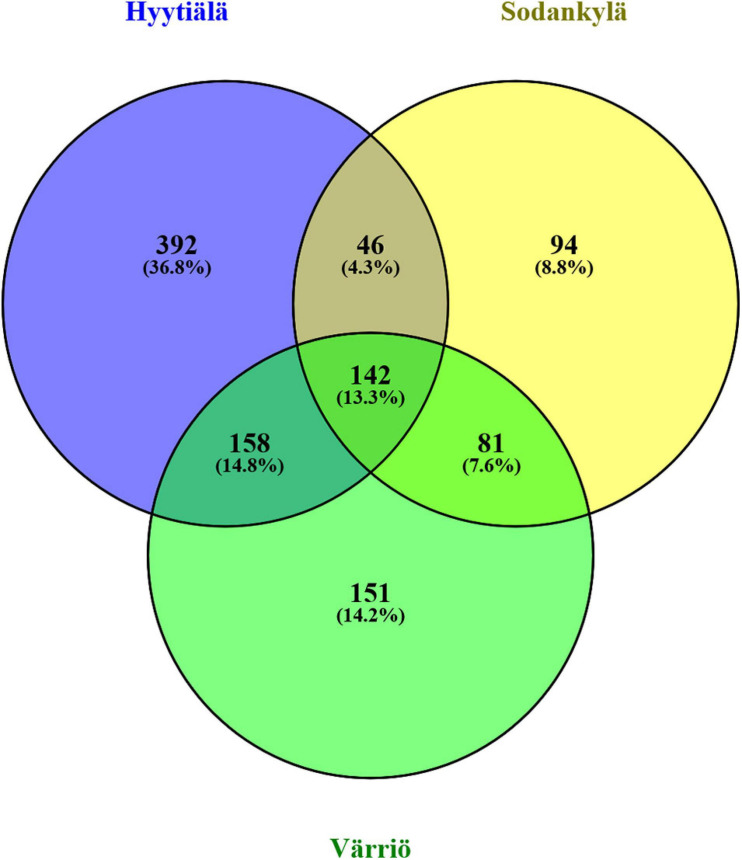
Venn diagram showing the unique and shared OTUs in the three locations between Hyytiälä (southern), Sodankylä (northern), and Värriö (subarctic) boreal pine forests. The number of total OTUs obtained from the three locations was 1,064.

The three locations shared 13.3% of the OTUs (142 OTUs). Hyytiälä harbored the highest percentage of unique OTUs (36.8%, 392 OTUs), followed by Värriö (14.2%, 151 OTUs) and Sodankylä (8.8%, 94 OTUs). A high percentage of unique OTUs indicated site-specific fungal communities (Figure 3).

**FIGURE 4:**
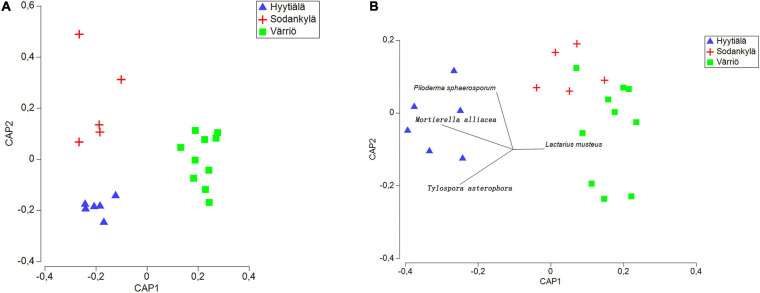
Canonical analysis of principal (CAP) coordinates showing the fungal community structure **(A)** and functional structure **(B)** in the three locations between Hyytiälä (southern), Sodankylä (northern) and Värriö (subarctic) boreal pine forests. The list of the top 10 most abundant species was used as the explanatory variable in the functional structure. Species not shown are not significantly correlated with fungal communities.

The three locations formed distinct fungal communities (Figure 4A), and subsequent PERMANOVA confirmed the differences among the fungal communities (*P* < 0.05 for each of the pair).

### Fungal Community Composition of Predicted Function in the Three Locations

In total, 490 OTUs (46.1% of the total OTUs) and 132,665 sequences (71.3% of the total sequence) were assigned to the three trophic modes. Of these, the symbiotroph mode accounted for 81.5% of the sequences, followed by the saprotroph mode at 5.8% and the pathotroph mode at 0.1%.

**FIGURE 5:**
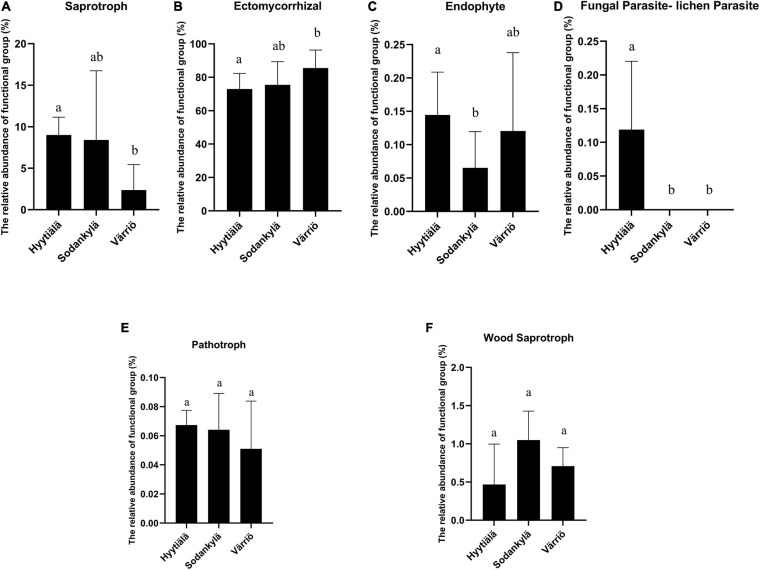
The functional groups showing significant differences **(A–D)** and commonly presented **(E,F)** in the three locations between Hyytiälä (southern), Sodankylä (northern) and Värriö (subarctic) boreal pine forests. **(A)** saprotroph, **(B)** ectomycorrhizal, **(C)** endophyte, **(D)** fungal parasite-lichen parasite s, **(E)** pathotroph, and **(F)** wood saprotroph.

The Värriö site displayed a higher abundance of symbiotrophs, with the dominance of ectomycorrhizal fungi (Figure 5B), whereas Hyytiälä had a higher abundance of saprotrophs (Figure 5A) with a great presence of fungal parasites (lichen parasite and endophytes) (Figures 5C,D). Pathotroph and wood saprotroph were commonly present as the core functional groups in these three regions (Figures 5E,F). The canonical analysis of principal (CAP) showed that only two fungal functional communities were formed, within which the functional structure present in Hyytiälä was separate from that in Sodankylä and Värriö (Figure 4B). DistLM analysis using the top 10 most abundant species as explanatory variables showed that *Lactarius musteus* was correlated with the functional structure in Sodankylä and Värriö, whereas *Piloderma sphaerosporum*, *Mortierella alliacea*, and *Tylospora asterophora* were linked to the functional structure in Hyytiälä (Figure 4B).

## Discussion

### Soil Fungal Diversity and Richness in the Three Locations

We carried out a comparative study aiming to give insight about the geographical link of fungal communities. The soil fungal diversity did not change across the three assessed locations, which is somewhat contrary to the previous results obtained on a larger scale, wherein the soil fungal diversity reached its highest values in temperate forests, and decreased in both the boreal forests and tropical forests (Shi et al., 2014). The results in our study might be explained by the fact that we remained at the local/regional scale. Within a certain range of scale, the soil fungal diversity in the forest with same tree species might remain the same despite of differences in other factors (e.g., soil properties). The southern boreal forest (Hyytiälä) harbored a greater fungal richness than the two northern forests (Sodankylä and Värriö). On a global scale, it has been confirmed that the richness of soil fungi decreases with increasing latitude, spanning from 18.25 °N to 53.29 °N and 109.62 °E to 124.70 °E (Hu et al., 2019). The diversity of microorganisms tends to increase as the latitude decreases (Hillebrand, 2004). Similarly, the latitude can also affect the richness of plant fungal endophytes in Scots pine needles (Terhonen et al., 2011). This difference in terms of latitude may be indirectly caused by changes in site characteristics, climate and humidity. Previous studies have shown that few fungi can be considered psychrophilic (Baxter and Illston, 1980). The warmer climate in southern Finland would favor fungi growth, which can increase the soil fungal richness (Tang et al., 2015).

### Core Fungal Taxonomic Groups in the Three Locations

The abundances of Basidiomycota and Ascomycota did not differ across the three locations, respectively. In the boreal forest soils, the fungal communities were dominated by Basidiomycota (66.5%) and Ascomycota (12.2%), which was consistent with the previous study carried out on a global scale (Tedersoo et al., 2014). However, the abundance of Ascomycota (12.2%) in our study was much lower than that carried out on a global scale (31.3%) (Tedersoo et al., 2014). This might be due to the soil properties and local climate factors, which are both relevant to the key fungal communities (Thomson et al., 2015; Větrovský et al., 2019). Previous studies have demonstrated that most members of the Zygomycota genus are saprobic (White et al., 2006). Variations in soil temperature, moisture and evaporation from the north to the south might cause the increase in saprophytic fungi, and facilitate high rates of organic matter decomposition (Chatterjee et al., 2008). The colder and less rainy northern conditions presumably caused reductions in the relative abundance of Zygomycota.

Across the three locations, the abundances of *Cortinarius*, *Piloderma*, and *Suillus* were not affected by the locations. All three genera belong to the functional group of ectomycorrhizal fungi, and have been reported to have a symbiotic relationship with *Pinus* spp. (Dahlberg and Finlay, 1999; Glowa et al., 2003; Teasdale et al., 2013). For example, *Cortinarius* is the largest genus of the ectomycorrhizal fungi, and has been found in *P. radiata* plantations, although it is relatively low in abundance (Teasdale et al., 2013). *Piloderma* has a wide range of possible hosts, and can increase soil nutrient availability via mineralization, thereby promoting the growth of coniferous forests (Glowa et al., 2003). *Suillus* has high host-specificity toward conifers, and its distribution is consistent with the natural distribution patterns of the northern hemisphere (Dahlberg and Finlay, 1999). The stability of the abundance of these three genera indicates that they may be the core fungal communities in the boreal forest. The changes in climate, temperature, precipitation and other factors caused by geographical location might have little impact on the levels of abundance of certain fungal generalists. On the contrary, the hosts can play more dominant roles in shaping fungal groups.

### Overall Soil Fungal Community Structure in the Three Locations

The existing evidence has indicated that fungi show certain regularities of behavior and unique biological patterns in different geographic gradients in forest soil (Talbot et al., 2014; Ma et al., 2017; Xavier de Lima et al., 2018). The fungal communities’ compositions and functions exhibited significant distance–decay relationships; that is, the fungal community’s structure may be more similar in a region close by than in one at a distance (Huang et al., 2020), suggesting a greater environmental difference in the niche. The three locations only shared a few OTUs, with relatively high numbers of unique OTUs in each, especially in Hyytiälä. This observation is in line with a previous study, which found that the soil fungal community in question became more divergent due to spatial factors (Chatterjee et al., 2008). These might explain the northern and subarctic locations had similar fungal functional structures compared to the southern location.

*Lactarius musteus* was positively correlated with the fungal functional structure in Sodankylä and Värriö sites, whereas *Piloderma sphaerosporum*, *Mortierella alliacea*, and *Tylospora asterophora* were positively correlated with the Hyytiälä site. The southern boreal forest has milder and rainier conditions than the northern sites, which is more conducive to the growth of saprophytic fungi (Chatterjee et al., 2008). Ectomycorrhizal symbiosis seemed to have evolved as an adaptation mechanism in the boreal forest, wherein the slow decomposition process of plant litter leads to nitrogen deficiency (Martin et al., 2016). This might partly explain the higher abundance of saprophytic functional groups at the southern site and the higher abundance of ectomycorrhizal fungi at northern sites. Endophytic fungi are essential for remedying nutrient leaching losses when rainfall intensity increases (Slaughter et al., 2016; Martínez-García et al., 2017; Lynch, 2019). The higher abundance of endophytic fungi in Hyytiälä might be a part of the plant–microbe response to environmental changes. The reason for this may be the relatively high amount of precipitation in the south, which could increase leaching. It is important to bear in mind that only half of the OTUs in our study was able to assign to functional groups. The results of the function prediction, therefore, can only reflect the fungal community function partially. Moreover, the current study only included single site in each location, which possibly hinder the interpretation of observation. Further studies with high number of study sites are necessary to draw clear picture of microbial community in regional scale.

## Conclusion

The soil fungal diversity did not differ among the three locations and the fungal species richness, however, decreased from the southern to the northern and subarctic boreal forests in Finland. Across the three locations, Basidiomycota and Ascomycota were the dominant phyla, and *Cortinarius*, *Piloderma* and *Suillus* were the core fungal genera, whereas, the pathotroph and wood saprotroph were commonly present in the boreal Scots pine forest. The similarity of fungal functional structures in the northern and subarctic forests suggests a distance–decay relationship in fungal communities and functions associated with geographic location.

## Data Availability Statement

Publicly available datasets were analyzed in this study. This data can be found here: The sequence data are available from the Sequence Read Archive (SRA) of the National Center for Biotechnology Information (NCBI) under project accession no. PRJNA703504.

## Author Contributions

JP, JH, and HS: conceptualization. HS, MS, and Z-LQ: methodology and writing—original draft preparation. HS and Z-LQ: software, formal analysis, and data curation. Z-LQ: validation. JH, JP, KK, and FB: investigation. MS, JP, KK, FB, JH, and HS: writing—review and editing. JH, JP, KK, and HS: funding acquisition. All authors have read and agreed to the published version of the manuscript.

## Conflict of Interest

The authors declare that the research was conducted in the absence of any commercial or financial relationships that could be construed as a potential conflict of interest.

## Footnotes

1 http://www.ebi.ac.uk/ena/data/view/PRJEB21587

2 http://www.ebi.ac.uk/ena/data/view/PRJEB10726

## Supplementary Material

Supplementary Figure 1 Rarefaction curve showing the sequence depth and observed OTUs.

Supplementary Figure 2 Pie chart showing the overall distribution of fungal sequences (*n* = 186,138) at the phylum level across the three locations in boreal pine forests.

Supplementary Table 1 The basic information on the sites in the three locations.

Supplementary Table 2 The soil properties and plant vegetation information in the two northern locations.
