# Supplementary material for: Soil Fungal Community Structure in Boreal Pine Forests: From Southern to Subarctic Areas of Finland
Source: Front Microbiol. 2021 May 26;12:653896. doi: 10.3389/fmicb.2021.653896 (PMC8188478; doi:10.3389/fmicb.2021.653896)
Supplement: Supplementary Figure 1 — Rarefaction curve showing the sequence depth and observed OTUs. [file Table_1.DOCX]

**Supplementary Figure:**

**Figure S1.** Rarefaction curve showing the sequence depth and observed OTUs.

**
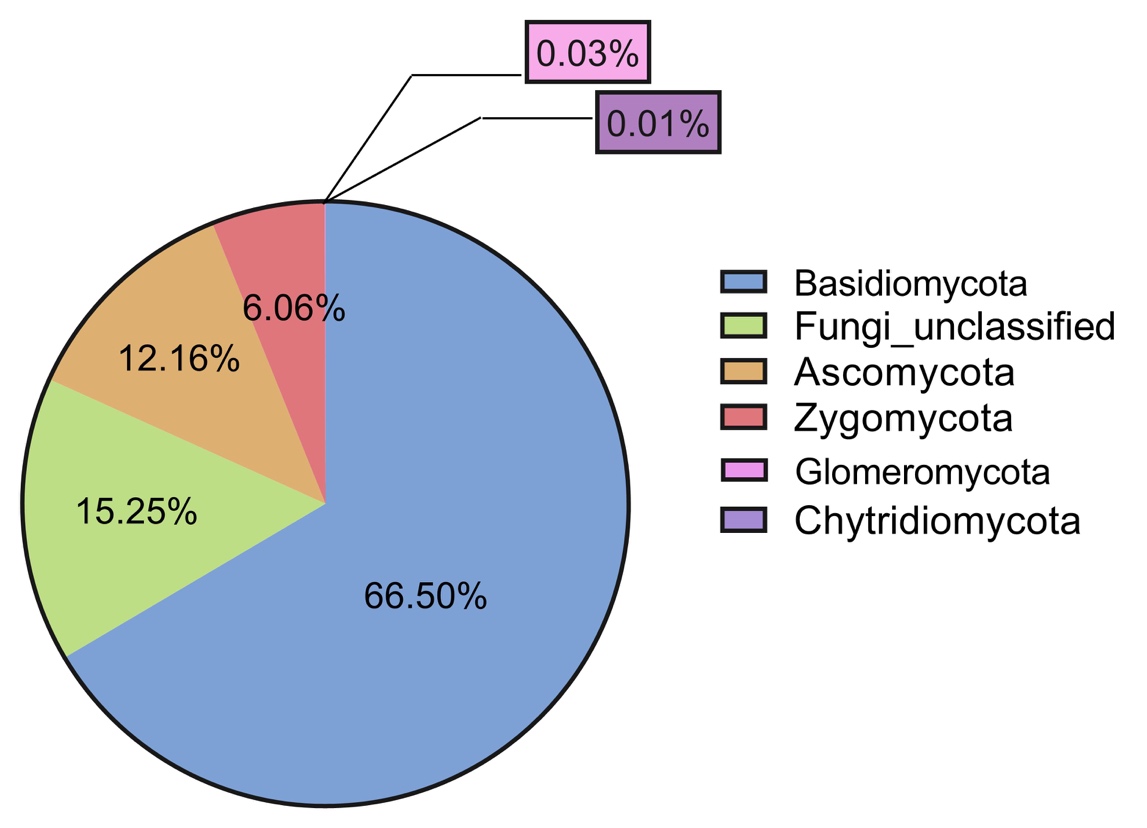
**

**Figure S2.** Pie chart showing the overall distribution of fungal sequences (n = 186,138) at the phylum level across the three locations in boreal pine forests.

**Table S1.** The basic information on the sites in the three locations.

**Table S2^*^.** The soil properties and plant vegetation information in the two northern locations

^*^The data were obtained from Santalahti et al. (2018). Unfortunately, such data on the southern site were not available.
